# Correlation between Frequency of Eating Out of Home and Dietary Intake, Sleep, and Physical Activity: A Survey of Young CDC Employees in China

**DOI:** 10.3390/ijerph19063209

**Published:** 2022-03-09

**Authors:** Yu Zhang, Xianglai Sang, Yibo Wu, Tuo Liu, Ran Niu, Lu Han, Qi Wang, Xiaocheng Liang

**Affiliations:** 1 Chinese Center for Disease Control and Prevention, Beijing 102206, China; hanlu@chinacdc.cn; 2 National Institute for Occupational Health and Poison Control, Chinese Center for Disease Control and Prevention, Beijing 100050, China; liutuo@niohp.chinacdc.cn; 3 Gansu Center for Disease Control and Prevention, Lanzhou 730000, China; sangxianglai@163.com (X.S.); lxc201@aliyun.com (X.L.); 4 School of Public Health, Peking University, Beijing 100871, China; 5 National Institute for Nutrition and Health, Chinese Center for Disease Control and Prevention, Beijing 100050, China; niuran@ninh.chinacdc.cn; 6 Xinjiang Center for Disease Control and Prevention, Urumqi 830002, China; wangqi1@chinacdc.cn

**Keywords:** young employees, CDC, eating out of home, dietary intake, sleep, physical activity

## Abstract

Objectives: We aimed to investigate the correlation between the frequency of eating out of home and dietary intake, sleep, and physical activity among young employees from the Center for Disease Control and Prevention in China. Methods: Using the cluster sampling method, 6099 employees aged 40 years or below from the Center for Disease Control and Prevention (CDC) from 32 provinces in China were interviewed using an online questionnaire survey. The frequency of eating out of home, dietary intake, sleep, and physical activity of all participants was described, and correlation analysis was used to study the relationships between eating out of home frequency and related indicators. Results: A total of 5353 valid questionnaires were collected with the recovery rate of 87.77%. The results show that 85.8% of participants eat out of home one to five times per week, 10.1% eat out of home more than six times, and 4.1% never eat out. Correlation analysis showed that eating out of home is negatively correlated with a daily vegetable and fruit intake. The lower the intake of vegetables and fruits, the more obvious this tendency. Eating out of home is positively correlated with a daily intake of meat as well as a weekly intake of aquatic products. The higher the intake of meat and aquatic products, the more obvious this tendency. There was a negative correlation between eating out of home and sleep duration and physical activity. The lower the duration of sleep and physical activity, the more obvious this tendency. Conclusions: Based on existing survey data, young employees from the CDC eat out of home regularly, which may affect dietary intake, sleep, and physical activity. Targeted health education programs are urgently needed to assist in the promotion of a healthy lifestyle and reduce the potential risk of chronic disease.

## 1. Introduction

Eating out of home has become increasingly popular in many countries [1]. With the continuous change in lifestyle in China, the proportion of young people eating out of home has also increased year by year [2]. 

Eating out of home options are usually high in energy and fat but low in other nutrients [3,4,5,6,7]. In the U.S., percentages of calorie intake attributed to eating out of home have significantly increased [4]. Eating on the run was associated with higher intakes of soft drinks, fast food, total fat, and saturated fat, and a lower intake of several healthy foods [8]. This less-than-optimal nutritional profile has raised public health concerns regarding the impact of eating out on long-term wellbeing [9].

At present, a growing number of studies suggest that eating out of home behavior may be a risk factor for obesity and chronic noncommunicable diseases [10,11]. Sleep and physical activity time are also strongly associated with chronic noncommunicable diseases [9]. Some studies provided evidence that certain dietary patterns and foods may have an impact on sleep quality [12]. Eating behavior may also be related to physical activity time [13]. Studying the relationships between eating out and sleep and physical activity can provide a clearer picture of the overall risk factors for chronic noncommunicable diseases. 

The tendency to eat out of home appears to be higher among physicians and other medical employees, especially younger ones, due to factors such as heavy workload and promotion pressure [14,15]. In particular, young adults lack the time to sit down and eat a meal at home [9]. The main objective of this study was to describe the frequency of eating out of home among young employees from the Center for Disease Control and Prevention, and to analyze the correlation between eating out of home and dietary intake, sleep, and physical activity. These data can help us develop targeted health education programs to reduce the risk of related diseases.

## 2. Materials and Methods

### 2.1. Subjects

A total of 6099 employees aged 20 to 40 years from the Center for Disease Control and Prevention (CDC) from 32 provinces in China were recruited to participate in this survey. The survey period was from October to November 2019. The protocols used in this study were approved by the Ethical Committee of the Chinese Center for Disease Control and Prevention (No. 201928). Informed consent was obtained for all respondents.

### 2.2. Questionnaire Design and Survey Methods

We designed the questionnaire based on survey needs and previous research and contained 72 questions divided into four dimensions, including basic information, ideological status, emotions, and health-related behavior [16,17], which were described in our previous study [18]. Eating out of home behavior included restaurants, canteens, takeout, and roadside stalls, according to the definition by Orfanos et al. [19] in 2007. If the average number of eating outside of the home is less than once per day, it was not regarded as eating out of home behavior. For dietary intake, we referred to the Dietary Guidelines for Chinese Residents (2016) [20], which contain the recommended quantities of various diets. Physical activity levels were measured in METs as recommended by the IPAQ Panel [21]. If the amount of exercise was less than three times per week for less than 30 min each, it was not considered physical activity.

In this study, the cluster sampling method was used to carry out a self-made online questionnaire survey and relevant data were collected online by scanning a two-dimensional code or logging into a survey link. Before the survey, the investigators were trained and instructed to fill in the form online. The questionnaire data were cleaned and coded by special personnel and the questionnaires with inconsistent, incomplete, and abnormal information were eliminated.

### 2.3. Statistical Analysis

The results are presented as mean values ± standard deviation (SD). The Kendall grade correlation coefficient test and a multinomial logistic regression analysis were used to analyze the relationships between variables. All of the statistical analyses were performed using Statistical Product and Service Solutions 13.0 software (International Business Machines Corporation, New York, NY, USA)and significance was set to an error rate of α = 0.05.

## 3. Results

### 3.1. Participant Characteristics

A total of 5353 valid questionnaires were included and used for statistical analysis, accounting for 87.77% of the total questionnaires: 1886 from men (35.23%) and 3467 from women (64.77%). A total of 1670 respondents (31.20%) were aged 18 to 30 years and 3683 (68.80%) were aged 31 to 40 years, as shown in Table 1. The average Body Mass Index (BMI) index was 22.5 ± 7.9 kg/m^2^.

### 3.2. Correlation between Eating Out of Home and Related Indexes

A total of 85.8% of participants reported eating out of home one to five times per week, 10.1% ate out of home more than six times, and 4.1% never ate out. Correlation analysis showed that eating out of home behavior had no correlation with the age of the participant. There was a correlation between eating out of home and sex (r = −0.060, *p* < 0.001), including that being a woman was associated with a higher frequency of eating out of home. There was also a correlation between eating out of home and BMI (r = 0.028, *p* = 0.029). Overweight and obese respondents were more likely to eat out of home, as shown in Table 2.

Eating out of home behavior had no correlation with the daily intake of vegetables. Meanwhile, there was a positive correlation between eating out of home and the weekly intake frequency of meat and aquatic products (r = 0.035 and 0.105, respectively, *p* < 0.01), and a negative correlation between eating out of home and the daily intake of grains and tubers and fruits (r = −0.033 and −0.041, respectively, *p* < 0.05), as shown in Table 3.

The results showed that the number of participants who slept for less than 6 h was 1612, accounting for 30.1%. We also found that eating out of home was negatively correlated with sleep duration and physical activity time (r = −0.050 and −0.075, respectively; *p* < 0.001). Eating out of home more often was associated with less sleep and physical activity time, as shown in Table 4.

### 3.3. Regression Analysis of Eating Out of Home and Related Indicators

A multinomial logistic regression analysis of the above variables showed that the tendency of men to choose to eat out of home one to five times per week was lower than that of women. Compared to those who never ate out of home, the tendency for those aged 18 to 30 years to choose to eat out of home more than six times per week was 2.019 times that of those aged 31 to 40 years (*p* < 0.001). There was no statistically significant difference in the propensity of different sexes to choose to eat out of home more than six times per week. The results of the correlation test differed: there was no statistically significant difference in the propensity of people with different BMI ratings to choose to eat out of home compared to those who never ate out of home (Table 5).

Compared to those who never ate out of home, those with a vegetable intake of 300–500 g were more likely to choose to eat out of home 1–5 times per week, 2.007 times that of the group with a vegetable intake of more than 500 g: the lower the vegetable intake, the higher the tendency. The group with a fruit intake of 100–200 g was more inclined to choose to eat out of home 1–5 times per week, a tendency 3.473 times higher than that of people who did not eat fruit. Therefore, the lower the fruit intake, the higher the tendency. Those who consumed less than 50 g of meat were more inclined to choose to eat out of home one to five times per week, a tendency that was 2.204 times that of those who did not eat meat. These results showed that the higher the intake of meat, the more obvious this tendency. Those who ingested aquatic products were more inclined to choose to eat out of home one to five times per week. The tendency of less than three times per week was 2.531 times that of those who did not eat aquatic products. The higher the aquatic product intake, the more obvious this tendency (Table 6).

Compared to those who never ate out of home, those who slept 7 h per day were more likely to choose to eat out of home one to five times per week, a tendency that was 2.160 times that of those who slept more than 8 h. People with less than 30 min of physical activity per day were more likely to choose to eat out of home one to five times per week. This tendency was 1.569 times that of people with more than 30 min of physical activity. The lower the physical activity time per day, the more obvious this tendency. Compared to people who ate out of home more than six times per week, people who slept less were more likely to choose to eat out of home six times per week. People who slept for 7 h were 1.961 times more likely to choose to eat out of home than those who slept for more than 8 h. The tendency to eat out of home for those who slept less than 6 h was 2.404 times that of those with more than 8 h of sleep. There was no statistically significant difference in the tendency of people to choose to eat out of home more than six times among groups with different physical activity durations (Table 7).

## 4. Discussion

Relevant studies claim that there is a certain correlation between sleep time, exercise time, and eating behavior, and good eating behavior can promote sleep and improve motivation to exercise [13,14,22]. Therefore, it is of great importance to understand the eating out of home status of specific occupational populations, such as CDC employees, and its influence on dietary intake and other health-related factors.

In our previous study, we found that the CDC staff are under great pressure and often work overtime [18], so they tend to eat out more. Meanwhile, food from restaurants and takeout that contain high oil, sugar, and fat increase energy intake.

The dietary intake survey showed that eating out of home behavior was positively correlated with an increased intake of meat and aquatic products and negatively correlated with an intake of cereals, tubers, vegetables, and fruits. Compared to those who never ate out of home, the higher the consumption of meat and aquatic products, the more obvious this tendency. The lower the intake of vegetables and fruits, the more obvious this tendency. For those who ate out of home more than six times per week, the proportion of meat intake over 75 g was 44.7%, which exceeds the recommended dietary intake of Chinese residents. A unanimous conclusion was obtained in the survey conducted by Chen et al. [23] on nonheavy physical labor occupations in China. Their survey suggested that the obesity associated with eating out was not caused by an increase in carbohydrates, but rather by the fat in meat and the sugar in food. However, this kind of dietary pattern negatively affects cardiovascular health [24]. Data from the EPIC study of 10 European countries showed that eating out of home significantly reduced the amount of fruit and vegetables consumed and significantly increased the possibilities of energy intake and sedentary lifestyle [20]. In our study, we also found that vegetable intake correlated with eating out of home, and that the lower the vegetable intake, the higher the tendency to choose to eat out of home.

Excessive frequency of eating out of home may be associated with less sleep among young employees. Previous studies found that medical workers in seven cities in China were generally sleep-deprived due to work pressure, diet, and other reasons [25]. In our study, 30.1% of the participants slept less than 6 h, showing that the lower the duration of sleep, the greater the tendency to eat out of home. Rocío Barragán et al. surveyed 179 adults aged 20 to 73 for a two-week sleep evaluation and found that poor sleep patterns and eating behavior traits were correlated [22]. In our study, we also found that sleep duration was negatively correlated with the frequency of eating out of home, and 38.6% of those eating out of home more than six times slept less than 6 h compared to those who never ate out of home. The higher the intake of meat and aquatic products, the more obvious this tendency. Compared to those who never ate out of home, the lower the sleep duration, the more obvious this tendency. Eating out at dinner time may interfere with normal sleep schedules, leading to a late return home. Eating too much meat, fat, and oil can also prolong digestion, which can lead to late sleep or poor-quality sleep and affect the sleep duration [12]. Chronic sleep deprivation can lead to mental fatigue, poor stress tolerance, job burnout, and an increased risk of chronic disease [18,26,27]. In a study of 40 overweight and obese adolescents in 2018, Andrea B Goldschmidt et al. [28] found that a regular diet, in addition to a reduced meat and dessert intake at dinner, significantly improved sleep quality. Poor eating habits may gradually become an involuntary behavior [19] and affect the future health of young employees. Therefore, health education on dietary behavior, especially advocating for eating at home as much as possible, is very important for young employees from the CDC.

Eating out of home may also affect the duration of and willingness to participate in physical activity. In our study, we found that the high tendency to eat out of home correlated with a low physical activity time. The lower the duration of physical activity, the more obvious this tendency. Previous studies in China found that there was insufficient exercise time among medical workers [25], which was associated with a higher prevalence of chronic diseases among young medical workers. In a study conducted in Morocco, it was found that eating behavior can affect physical activity; more than 50% of adolescents who enjoyed desserts, snacks, and takeout were more likely to sit in front of the TV than engage in outdoor exercise [29].

Previous studies found that people who were more physically active tended to eat more vegetables and fruits [30]. Our study also showed a clear correlation between vegetable intake and eating out of home. The lower the vegetable intake, the higher the tendency to eat out of home. People who ate out of home more frequently had a lower fruit intake and their physical activity frequency was also relatively low. We found that 35.1% of those who ate out of home more than six times per week performed no physical activity, while 35.7% of those who did not eat out of home often exercised more than 30 min per day. The correlation might be reflected in the fact that people who eat out of home for a long time tend to be less conscious of their health and are less likely to deliberately choose to exercise. A study conducted by Liu YB et al. [31] on the correlation between health beliefs and exercise among 4500 older adults in China reached similar conclusions. They suggested that a correlation exists between eating behavior and physical activity, which is determined by health literacy. Another possibility is that people who eat out of home more often tend to suffer from more work and life stress or higher levels of fatigue, and have less energy and willingness to participate in physical activity. This phenomenon was also found in our previously published article [19], where we found that young practitioners from the CDC had varying degrees of problems in diet, exercise, and sleep, which were affected by work pressure and job burnout. A study in the USA for the relationship between diet, physical activity, and weight also found that some obese people ate fast food because of work and they did not have enough time to exercise [32]. From the perspective of health promotion, eating out of home increases energy intake and the risk of obesity, so it is appropriate to increase the amount of physical activity. However, our survey results showed that, particularly among young professionals, eating out of home was negatively correlated with the amount of physical activity. This is an adverse phenomenon that deserves our vigilance.

## 5. Conclusions

Eating out of home behavior has a certain impact on dietary intake, sleep, and physical activity, and excessive eating out of home frequency increases the factors that are detrimental to health. There may be some bias in this study due to sample size, which may have affected the accuracy and credibility of the results. We acknowledge that we used an online questionnaire survey to detect the relationships between eating behavior and dietary intake, sleep, and physical activity. We did not have objective assessments of the investigated variables and no information on the validation of the questions used to collect this information is available. Despite some limitations, our findings represent a significant step forward, especially for determining possible correlations and suggesting targeted solutions.

## Figures and Tables

**Table 1:** Basic information of respondents.

	Cases (n)	Percentage (%)
Age (years)		
18–30	1670	31.2
31–40	3683	68.8
Sex		
Male	1886	35.2
Female	3467	64.8
Education level		
Bachelor degree or above	3083	57.6
Postgraduate degree	2270	42.4
Marital status		
Married	3907	73.0
Single	1348	25.2
Other situations	98	1.8

**Table 2:** Distribution and correlation of eating out of home in different groups.

	Never		1 to 5 Times per Week		≥6 Times per Week		r Value	*p* Value
	Cases (n)	Percentage (%)	Cases (n)	Percentage (%)	Cases (n)	Percentage (%)		
Age (years)							−0.018	0.180
18–30	51	23.1	1413	30.8	206	38.1		
31–40	170	76.9	3178	69.2	335	61.9		
Sex							−0.060	0.000
Male	98	44.3	1564	34.1	224	41.4		
Female	123	55.7	3027	65.9	317	58.6		
BMI							0.028	0.029
Thin	10	4.5	338	7.4	48	8.9		
Normal	140	63.3	2822	61.5	299	55.3		
Overweight	43	19.5	994	21.7	111	20.5		
Obesity	28	12.7	437	9.5	83	15.3		

**Table 3:** Correlation between eating out of home and dietary intake.

Intake per Day	Never		1 to 5 Times per Week		≥6 Times per Week		r Value	*p* Value
	Cases (n)	Percentage (%)	Cases (n)	Percentage (%)	Cases (n)	Percentage (%)		
Grains and tubers							−0.033	0.015
Never	14	6.3	21	0.5	32	5.9		
<250 g	60	27.1	1731	37.7	315	58.2		
250–400 g	22	10.0	2100	45.7	128	23.7		
>400 g	125	56.6	739	16.1	66	12.2		
Vegetables							−0.011	0.394
<300 g	141	63.8	3312	72.1	406	75.0		
300–500 g	63	28.5	1169	25.5	113	20.9		
>500 g	17	7.7	110	2.4	22	4.1		
Fruits							−0.041	0.002
Never	36	16.3	130	2.8	51	9.4		
<200 g	126	57.0	2981	64.9	349	64.5		
200–350 g	59	26.7	1480	32.2	141	26.1		
Meat							0.035	0.006
Never	15	6.8	36	0.8	13	2.4		
<40 g	66	29.9	828	18.0	80	14.8		
40–75 g	81	36.7	2121	46.2	206	38.1		
>75 g	59	26.7	1606	35.0	242	44.7		
Aquatic products(per week)							0.105	0.000
Never	390	8.5	88	16.3	60	27.1		
≤3 times	3813	83.1	405	74.9	145	65.6		
≥4 times	387	8.4	48	8.9	16	7.2		

**Table 4:** Correlation between eating out of home and sleep and physical activity.

	Never		1–5 Times per Week		≥6 Times per Week		r Value	*p* Value
	Cases (n)	Percentage (%)	Cases (n)	Percentage (%)	Cases (n)	Percentage (%)		
Sleep duration							−0.050	0.000
≤6 h per day	76	34.4	1327	28.9	209	38.6		
7 h per day	128	57.9	3119	67.9	315	58.2		
≥8 h per day	17	7.7	145	3.2	17	3.1		
Physical activity time							−0.075	0.000
Never	49	22.2	1150	25.0	190	35.1		
≤30 min per day	93	42.1	1920	41.8	195	36.0		
>30 min per day	79	35.7	1521	33.1	156	28.8		

**Table 5:** Multiple logistic regression analysis of the relationship between eating out of home and sex, age, and BMI.

Frequency of Eating Out of Home	Variables		Wald	*p* Value	Exp(B)	95% CI
1–5 times per week	Age	18–30	3.846	0.050	1.340	1.000–1.796
		31–40	Ref.	Ref.	Ref.	Ref.
	Sex	Male	10.893	0.001	0.640	0.491–0.834
		Female	Ref.	Ref.	Ref.	Ref.
	BMI	Thin	2.318	0.128	1.719	0.856–3.452
		Normal	0.654	0.419	1.179	0.791–1.758
		Overweight	3.304	0.069	1.624	0.963–2.74
		Obesity	Ref.	Ref.	Ref.	Ref.
≥6 times per week	Age	18–30	17.062	0.000	2.019	1.447–2.819
		31–40	Ref.	Ref.	Ref.	Ref.
	Gender	Male	0.816	0.366	0.865	0.631–1.185
		Female	Ref.	Ref.	Ref.	Ref.
	BMI	Thin	1.174	0.279	1.533	0.708–3.317
		Normal	1.516	0.218	0.748	0.471–1.188
		Overweight	0.006	0.937	0.976	0.531–1.794
		Obesity	Ref.	Ref.	Ref.	Ref.

**Table 6:** Multiple logistic regression analysis of the relationship between eating out of home and dietary intake.

Frequency of Eating Out of Home	Variables		Wald	*p* Value	Exp(B)	95%CI
1–5 times per week	Cereals	Never	0.094	0.759	0.949	0.677–1.33
		Yes	Ref.	Ref.	Ref.	Ref.
	Vegetables	≤300 g	19.238	0.000	3.430	1.977–5.950
		300–500 g	5.917	0.015	2.007	1.145–3.518
		≥500 g	Ref.	Ref.	Ref.	Ref.
	Fruits	≤100 g	32.146	0.000	3.553	2.292–5.507
		100–200 g	26.130	0.000	3.473	2.155–5.598
		Never	Ref.	Ref.	Ref.	Ref.
	Meat	≤50 g	5.091	0.024	2.204	1.109–4.379
		50–100 g	14.576	0.000	3.817	1.919–7.593
		≥100 g	19.427	0.000	4.820	2.395–9.701
		Never	Ref.	Ref.	Ref.	Ref.
	Aquatic product	≤3 times	31.670	0.000	2.531	1.832–3.497
		≥4 times	10.790	0.001	2.627	1.476–4.676
		Never	Ref.	Ref.	Ref.	Ref.
≥6 times per week	Cereals	Never	3.946	0.047	1.468	1.005–2.144
		Yes	Ref.	Ref.	Ref.	Ref.
	Vegetables	≤300 g	7.343	0.007	2.494	1.288–4.831
		300–500 g	0.220	0.639	1.177	0.595–2.328
		≥500 g	Ref.	Ref.	Ref.	Ref.
	Fruits	≤100 g	2.222	0.136	1.466	0.886–2.425
		100–200 g	0.329	0.566	1.175	0.677–2.041
		Never	Ref.	Ref.	Ref.	Ref.
	Meat	≤50 g	0.017	0.896	0.947	0.418–2.143
		50–100 g	2.217	0.137	1.850	0.823–4.160
		≥100 g	9.329	0.002	3.569	1.578–8.074
		Never	Ref.	Ref.	Ref.	Ref.
	Aquatic product	≤3 times	4.052	0.044	1.490	1.011–2.197
		≥4 times	2.092	0.148	1.634	0.840–3.179
		Never	Ref.	Ref.	Ref.	Ref.

**Table 7:** Multiple logistic regression analysis of the relationship between eating out of home and sleeping and physical activity.

Frequency of Eating Out of Home	Variables		Wald	*p* Value	Exp(B)	95% CI
1–5 times per week	Sleep duration	≤6 h per day	4.323	0.038	1.764	1.033–3.013
		7 h per day	8.692	0.003	2.160	1.295–3.605
		≥8 h per day	Ref.	Ref.	Ref.	Ref.
	Physical activity	Never	20.617	0.000	2.007	1.486–2.711
		≤30 min per day	8.771	0.003	1.569	1.164–2.113
		>30 min per day	Ref.	Ref.	Ref.	Ref.
≥6 times per week	Sleep duration	≤6 h per day	6.145	0.013	2.404	1.202–4.811
		7 h per day	3.852	0.050	1.961	1.001–3.841
		≥8 h per day	Ref.	Ref.	Ref.	Ref.
	Physical activity	Never	1.496	0.221	1.244	0.877–1.765
		≤30 min per day	0.013	0.908	0.979	0.688–1.394
		>30 min per day	Ref.	Ref.	Ref.	Ref.

## Data Availability

The data that support the findings of this study are available from the corresponding author, Y.Z., upon reasonable request.
